# Conservative approach for intra-amniotic *Candida albicans* colonisation. Case report and review of current evidence

**DOI:** 10.1515/crpm-2024-0047

**Published:** 2025-06-11

**Authors:** Beatriz Bové, Irene Barragán, Laia Pratcorona, Roser Porta, Raül de Diego, Ma Carmen Comas, María Méndez, Carlos Rodrigo Gonzalo de Liria

**Affiliations:** Department of Obstetrics and Gynaecology, Hospital Universitari Germans Trias i Pujol, Badalona, Barcelona, Spain; Department of Pediatrics, Hospital Universitari Germans Trias i Pujol, Badalona, Spain

**Keywords:** *Candida albicans*, intra-amniotic colonisation, chorioamnionitis, cervical cerclage, fluconazole, amphotericin

## Abstract

**Objectives:**

Intra-amniotic colonisation or infection caused by *Candida albicans* is rare. Given the shortage of reported cases, evidence on antifungal strategies and the choice of type and timing of delivery is limited.

**Case presentation:**

We report a case of intra-amniotic colonisation by *C. albicans* in a pregnant woman with a previous history of cervical cerclage and candidal vaginosis at 25 weeks of gestational age (GA). The diagnosis was made following preterm premature rupture of membranes at GA 28 weeks and 6 days. Treatment was started with amphotericin B and was replaced by fluconazole due to an anaphylactic reaction. The persistence of *C. albicans* in the amniotic fluid after 24 days of treatment prompted the decision to plan an elective caesarean delivery at 32 weeks’ GA. The infant showed no signs of fungaemia and had an uneventful clinical course after 14 days of treatment with fluconazole.

**Conclusions:**

Conservative management with intravenous fluconazole in pregnant women with intra-amniotic colonisation by *C. albicans* at early GA, can contribute to the prolongation of pregnancy while protecting the foetus from fungal disease.

## Introduction

Vaginal colonisation and symptomatic vulvovaginitis (VV) caused by *Candida* species are common conditions during pregnancy, affecting approximately 20–40 % of pregnant women. However, the incidence of complications is very low and usually arises in recurrent episodes of VV [1], 2]. Reported complications include chorioamnionitis, which occurs in 0.3–0.8 % of cases and is associated with adverse perinatal outcomes, primarily related to prematurity [3], [4], [5], [6]. This condition increases the risk of invasive candidiasis in neonates, which may raise mortality rates to 71 % and neurodevelopmental impairment rates to 86 % [5].

*Candida albicans* is the species most frequently implicated in VV and in intra-amniotic infection (IAI), spreading primarily via ascending route through the genital tract. This occurs in most cases after preterm premature rupture of membranes (PPROM), or due to the fungus’s capacity to cross intact foetal membranes, particularly following invasive medical procedures such as cervical cerclage, amniocentesis (AC), or *in vitro* fertilisation. Cases of haematogenous dissemination or retrograde translocation from the Fallopian tube, originating in the peritoneal cavity, have also been described [2], 5].

Microbial invasion of the amniotic cavity (MIAC) by *Candida* spp., representing the colonisation stage prior to IAI, poses a therapeutic challenge due to the limited and controversial evidence available. Similar to bacterial MIAC, early detection before the onset of an inflammatory response is thought to provide an opportunity to improve maternal-foetal outcomes through targeted antifungal therapy [7].

We report a case of a pregnant woman with MIAC caused by *C. albicans,* managed with intravenous antifungal therapy and guided by an interdisciplinary consensus involving the obstetrics, neonatology, and microbiology teams.

## Case presentation

A 26-year-old pregnant woman with no significant medical history, presented at 24 weeks’ gestational age (GA) with scant vaginal bleeding and suprapubic pain. The case was initially assessed as threatened preterm labour (TPL) following the identification of a partially dilated cervix, irregular asymptomatic uterine contractions, and a cervical length (CL) of 3 mm. Genital cultures were obtained, and urinalysis revealed pathological findings, while blood tests showed normal values. A single dose of fosfomycin was administered, along with a course of betamethasone for foetal lung maturation, magnesium sulphate for foetal neuroprotection, and tocolysis. On the third day of admission, in the absence of uterine contractions or any abnormalities on cardiotocography, and with no further clinical changes, a 2 cm cervical dilation with membrane exposure was observed. The case was subsequently re-evaluated as cervical insufficiency.

An AC was performed, revealing normal glucose levels and negative results for Gram staining and culture. This was followed by a tertiary cerclage. The patient was discharged at 25 weeks’ GA with a CL of 11–14 mm. During two follow-up visits, the stability of the CL was confirmed; however, the review of previous vaginal cultures, which had identified *C. albicans,* had been omitted.

At 28 weeks and 6 days, the patient presented with PPROM. There were no clinical or analytical signs suggestive of IAI, such as fever, maternal or foetal tachycardia, purulent vaginal discharge, or leucocytosis. Uterine contractions were absent, and CL remained stable. A new AC was performed, alongside a second complete course of betamethasone, azithromycin, ampicillin, and gentamicin, in accordance with protocol. Analysis of the amniotic fluid revealed glucose levels of 28 mg/dL, the presence of yeast on Gram staining, and no detectable white blood cell count. Other inflammatory markers in amniotic fluid were not measured, as they are not part of our routine laboratory workup for PPROM or TPL.

The findings were interpreted as intra-amniotic colonisation, with IAI considered unlikely given the absence of pyrexia or other clinical signs, normal laboratory parameters, and normal glucose levels in amniotic fluid. Although active vaginal candidiasis at the time of cerclage placement may have contributed to MIAC, we considered that, given the four-week interval between the procedure and PPROM, together with the absence of clinical or biochemical evidence of IAI, the amniotic colonisation most likely occurred following membrane rupture rather than prior to it. The cerclage was therefore maintained due to cervical insufficiency, with the aim of preventing preterm birth and a conservative approach was adopted. Treatment was initiated with amphotericin B at 340 mg/day, which was subsequently replaced with intravenous fluconazole at 400 mcg/day due to an allergic reaction. During hospitalisation, the patient remained asymptomatic, with no uterine contractions, a stable CL, and normal blood test results. Upon reaching 31 weeks’ GA and completing two weeks of antifungal therapy, the AC was reassessed, revealing normal glucose levels and a negative Gram stain, although culture subsequently confirmed persistent *C. albicans*. Following multidisciplinary consensus, the pregnancy was electively delivered by caesarean section at 32 weeks’ GA, and the cerclage was removed.

**Figure 1:**
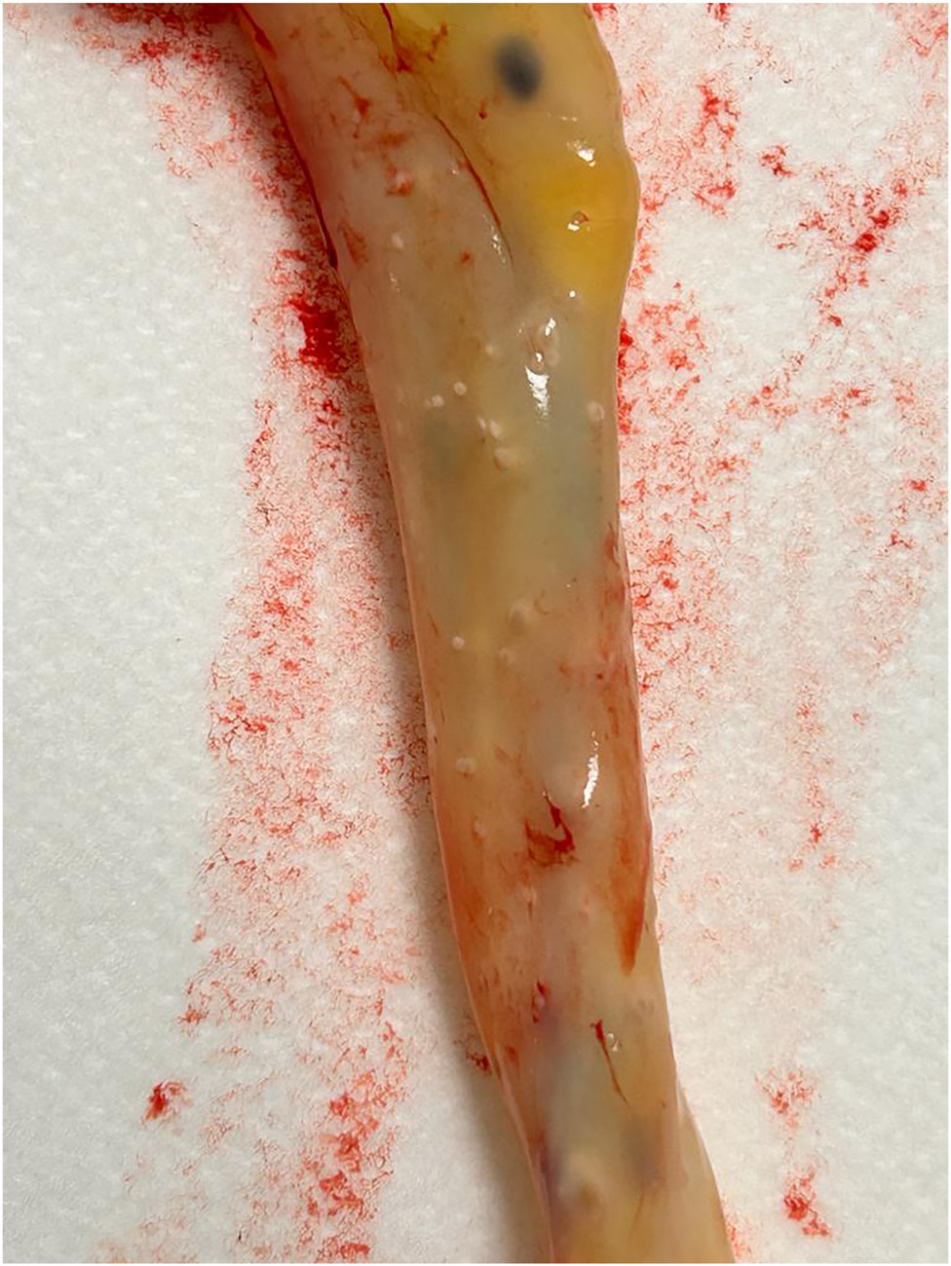
Umbilical cord showing funisitis lesions.

**Figure 2:**
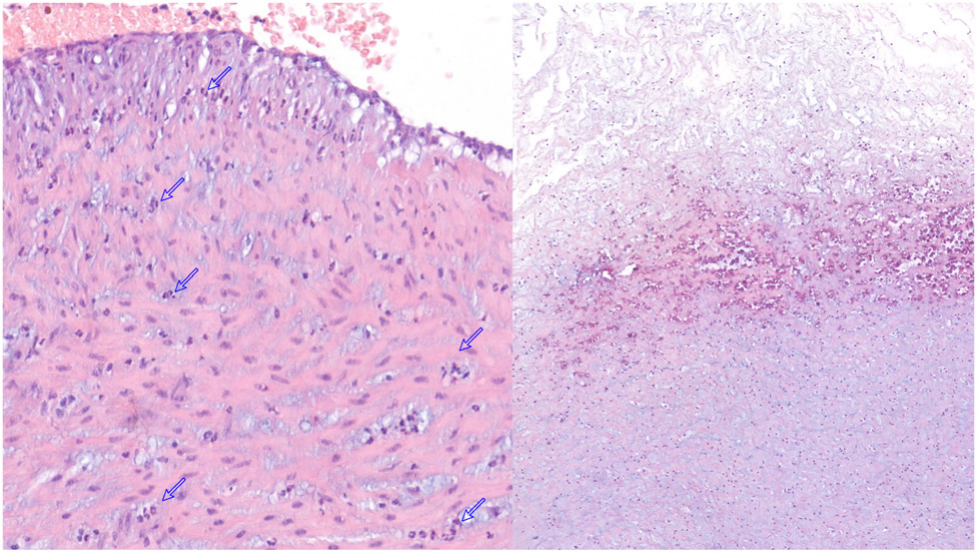
Placental acute chorioamnionitis: placental examination revealed a characteristic polymorphonuclear neutrophilic infiltration, consistent with acute chorioamnionitis. The neutrophilic infiltrate extended through the trophoblastic layer, invading both the fibrous chorion and amnion, with neutrophils forming small confluent clusters and microabscesses. No granulomas or fungal structures were identified microscopically within the amniotic surfaces. (A) Infiltration of the chorionic vessel wall by polymorphonuclear leukocytes (25×). (B) Calcifications and neutrophilic infiltration in Wharton’s jelly (5×).

A girl with an Apgar score of 9–10 was born, weighing 1,357 g, in good clinical condition, with a normal respiratory pattern and no skin lesions. Lesions in the umbilical cord compatible with funisitis were noted: rounded, whitish plaques of 1–2 mm distributed throughout the entire cord (Figure 1). The pathological examination of the placenta showed acute chorioamnionitis (Figure 2), and culture revealed a few colonies of *C. albicans* sensitive to fluconazole.

The colonisation cultures (axillary, inguinal, and rectal smear tests) were positive for *C. albicans*. The extended study showed no signs of systemic infection (negative blood and cerebrospinal fluid cultures). Cerebral and renal ultrasounds were normal, as was the ophthalmological examination. It was agreed to continue antifungal treatment with intravenous fluconazole at 6 mg/kg/day for 7 days, followed by 7 days of oral treatment. No signs of hepatotoxicity or other side effects were detected in laboratory tests. As additional measures, she was kept in an incubator without humidity, under strict contact isolation, and central catheters were avoided. The patient had a favourable outcome and remained asymptomatic and free of morbidity related to prematurity, except for anaemia requiring a blood cell transfusion in the third week of life.

She was discharged at 34 days of age. During follow-up visits until a corrected age of 9 months, she showed normal neurodevelopment.

## Discussion

### Obstetric management

In this case, we highlight the complexity of diagnosing and differentiating between MIAC and IAI caused by *C. albicans*. MIAC is defined as intra-amniotic colonisation without an associated inflammatory response or other clinical findings [7]. In contrast, IAI typically presents as a subclinical infection but may also manifest as miscarriage, cervical insufficiency, PPROM, or TPL. However, infection has also been reported as occurring secondary to PPROM or cervical insufficiency. In such scenarios, prolonged exposure of the membranes to vaginal flora may facilitate microbial invasion [3], 5]. In our case, both hypotheses regarding fungal invasion were considered: the presence of vaginal infection at the time of cerclage could have facilitated *C. albicans* invasion into the amniotic cavity, leading to PPROM, or the amniotic cavity could have been invaded following PPROM. Given the rupture of membranes without evidence of a significant inflammatory response, our initial diagnostic assessment was more consistent with intra-amniotic colonisation rather than IAI.

In addition, the diagnosis of IAI is further complicated because laboratory tests typically show no obvious signs of infection, and ultrasound findings are often limited to a single indicator: intra-amniotic sludge, which has been identified as a factor in recognising patients at risk of IAI [3], 5]. Consequently, the usual clinical and laboratory criteria for a presumptive diagnosis of IAI are often not met, and the condition is diagnosed only after the analysis of amniotic fluid. Definitive confirmation is established through characteristic histopathological findings on examination of the placenta and umbilical cord [2], 8].

Regarding the treatment of VV, recent studies indicate that vaginal colonisation by *Candida* spp. in pregnancy, whether asymptomatic or symptomatic, does not increase the risk of preterm birth or negatively affect pregnancy outcomes. However, in cases where VV due to *Candida* spp. is diagnosed, the predominant recommendation is the use of vaginal clotrimazole, owing to its potential protective effect in preventing preterm birth, which is attributed to its anti-inflammatory properties rather than its antifungal action [1], 9].

Systemic intravenous strategies or intra-amniotic injections have been proposed for the management of fungal intra-amniotic invasion. These interventions prolong gestation from 0 days to 8 weeks; however, it is important to consider that neonatal survival is less than 50 % [9]. Currently, intra-amniotic fluconazole is the most widely used antifungal agent despite its associated risks and limitations [10], [11], [12]. This drug crosses the placental membrane and, although there are reports of possible teratogenic effects, the most recent meta-analyses have not demonstrated a direct association with congenital anomalies, except for a potential risk of cardiac malformations following oral administration during the first trimester [13], [14], [15]. In addition, it has shown reduced activity against *Candida glabrata*, the second most prevalent species. Following intra-amniotic administration, foetal serum concentrations achieved are higher than maternal levels [5], 10]. Other studies have proposed amphotericin B as the preferred antifungal agent for the management of intra-amniotic colonisation in pregnant women, owing to its ability to cross the placenta without reported adverse effects on the foetus, as well as its greater efficacy against *C. glabrata.* The use of ketoconazole and micafungin has also been documented, although their efficacy and potential adverse effects remain uncertain [3], 5]. In our case, we opted for intravenous therapy rather than intra-amniotic instillation, based on evidence suggesting that maternal intravenous treatment achieves therapeutic levels in foetal circulation [10], rendering intra-amniotic administration unnecessary and potentially increasing the risk of toxicity and procedure-related complications [5], 10]. Therefore, our initial preference was to use a broader-spectrum antifungal agent with a lower resistance rate and a more favourable teratogenicity profile, which led us to initially opt for amphotericin B. However, following an anaphylactic reaction to this agent, and considering the gestational age, we regarded fluconazole as a safe and appropriate alternative therapeutic option.

Concerning MIAC, there is currently no established guidance on optimal management or timing of delivery [7]. For this reason, our decisions were guided by the available literature on Candida IAI. Current data indicate that once IAI is established, it typically progresses to spontaneous labour. In cases where this does not occur, antifungal therapy is initiated, antenatal corticosteroids are administered, and delivery is planned beyond 34 weeks’ GA. According to the limited evidence available on Candida IAI, in asymptomatic pregnant women under 34 weeks GA who are clinically and biochemically stable, the timing of delivery should be individualised [8], 16], 17]. Based on this, we opted for a conservative management approach. After two weeks of fluconazole treatment, *C. albicans* remained positive in the third AC. Consequently, following a comprehensive multidisciplinary risk–benefit assessment, the pregnancy was electively delivered by caesarean section.

### Neonatal management

The most typical form of candidal placental infection is funisitis. This case presented these characteristic lesions along the entire umbilical cord.

Congenital candidiasis in neonates may present with a broad clinical spectrum, ranging from isolated cutaneous involvement to disseminated systemic disease. Due to the rarity of reported cases and the limited body of available literature, establishing standardised management guidelines remains challenging. The primary risk for a foetus with intra-amniotic candidiasis is the development of congenital cutaneous candidiasis (CCC). CCC usually follows a benign course; however, in low birthweight preterm infants (<1,000 g), it presents a higher risk of progression to a systemic form, for which treatment with systemic antifungal agents is recommended [18], [19], [20].

The systemic form may or may not be accompanied by cutaneous manifestations and is typically followed by respiratory distress, sepsis, or meningitis. This form is particularly severe and affects preterm infants in greater proportion due to their immunosuppression and the non-integrity of mucocutaneous barriers. A mortality rate of 39–94 % has been reported. Early diagnosis and prompt treatment with antifungal agents are crucial [21].

First-line treatment is based on amphotericin B, with fluconazole as a second-line option when the former is contraindicated. The addition of caspofungin or micafungin to first-line treatment remains debated. Systemic treatment is recommended in neonates with suspected congenital candidiasis who meet the following criteria [21]: those presenting with respiratory distress, clinical or laboratory signs of sepsis, a birth weight of less than 1,500 g, exposure to broad-spectrum antibiotics, extensive instrumentation during delivery, invasive procedures in the neonatal period, positive blood cultures, or an underlying immune deficiency. There is ongoing debate regarding the treatment of CCC in term neonates, as they have a low risk of systemic spread. The decision to initiate treatment in our asymptomatic patient was guided by the presence of multiple risk factors, including prematurity, very low birthweight, extensive colonisation, and prolonged intrauterine exposure. The duration of antifungal treatment should be at least 10 days. As an additional recommendation, some authors have suggested avoiding humidification in incubators to prevent fungal proliferation, particularly in neonates weighing less than 1,000 g [2].

### Patient perspective

*“It has been a very difficult time for me and my husband, it was our first child. We have been helped enormously by the Hospital Germans Trias i Pujol. Our daughter was born very healthy, she did everything without help, she’s now 6 months old and she is very healthy. We look very positively on the right decisions made by the doctors.*”

## Conclusions

The management of pregnant women with MIAC or IAI caused by *C. albicans* is challenging and controversial, requiring close collaboration between obstetric and neonatal teams. Our case provides further evidence of the effectiveness of intravenous fluconazole in pregnant women with intra-amniotic *C. albicans* colonisation in reducing neonatal morbidity associated with both prematurity and systemic foetal infection.
